# Prior exposure to speech rapidly modulates cortical processing of high-level linguistic structure

**DOI:** 10.1093/cercor/bhag070

**Published:** 2026-07-01

**Authors:** Qingqing Meng, Yiwen Li Hegner, Iain Giblin, Catherine McMahon, Blake W Johnson

**Affiliations:** The HEARing CRC, Department of Audiology and Speech Pathology, The University of Melbourne, 550 Swanston St, Carlton, VIC 3053, Australia; Department of Cognitive Science, Macquarie University, Australian Hearing Hub, 16 University Avenue, Macquarie University, NSW 2109, Australia; National Acoustic Laboratories, Australian Hearing Hub, 16 University Avenue, Macquarie University, NSW 2109, Australia; The HEARing CRC, Department of Audiology and Speech Pathology, The University of Melbourne, 550 Swanston St, Carlton, VIC 3053, Australia; Department of Linguistics, Macquarie University, Australian Hearing Hub, 16 University Avenue, Macquarie University, NSW 2109, Australia; MEG Center, University of Tübingen, Otfried-Müller-Straße 47, 72076 Tübingen, Germany; Department of Linguistics, Macquarie University, Australian Hearing Hub, 16 University Avenue, Macquarie University, NSW 2109, Australia; The HEARing CRC, Department of Audiology and Speech Pathology, The University of Melbourne, 550 Swanston St, Carlton, VIC 3053, Australia; Department of Linguistics, Macquarie University, Australian Hearing Hub, 16 University Avenue, Macquarie University, NSW 2109, Australia; HEAR Centre, Macquarie University, Australian Hearing Hub, 16 University Avenue, Macquarie University, NSW 2109, Australia; The HEARing CRC, Department of Audiology and Speech Pathology, The University of Melbourne, 550 Swanston St, Carlton, VIC 3053, Australia; Department of Cognitive Science, Macquarie University, Australian Hearing Hub, 16 University Avenue, Macquarie University, NSW 2109, Australia

**Keywords:** brain imaging, magnetoencephalography, prior knowledge, speech intelligibility

## Abstract

Neural activity has been shown to track hierarchical linguistic units in connected speech, and these responses are modulated by changes in speech intelligibility resulting from spectral degradation. In this study, we manipulated prior knowledge to enhance the intelligibility of physically identical speech sentences and tested whether this improvement would strengthen neural tracking responses. Cortical magnetoencephalography responses were recorded from 23 normal-hearing participants while they listened to intelligible speech followed by either the same (matched) or different (unmatched) unintelligible speech. When prior knowledge was available, cortical coherence at higher-order linguistic rates, particularly phrase and sentence rates, was enhanced relative to the unmatched condition and was predominantly lateralized to the left hemisphere. In contrast, cortical coherence to word-level units, which aligned with acoustic onsets, was bilateral and did not show a significant modulation by contextual information. No such coherence enhancement was observed when unintelligible speech preceded intelligible speech. This dissociation suggests that cerebral tracking of linguistic information is directly influenced by intelligibility, which itself is strongly shaped by physical speech cues. These findings provide an objective and sensitive neural index of speech intelligibility and help explain why previous studies have reported no effect of prior knowledge on cortical entrainment.

## Introduction

A dramatic enhancement in the perceived intelligibility of distorted speech signals can be achieved by providing prior information on the content of the signal (Remez et al. 1981; Jacoby et al. 1988). This top-down perceptual change, referred to as perceptual “pop-out” (Davis et al. 2005), is invoked rapidly and reliably with immediate prior exposure to a clear speech signal.

Several neuroimaging studies have examined changes in brain activity associated with this perceptual “pop-out” effect. In a series of functional magnetic resonance imaging (fMRI) studies, decreased activations have been reported in the left superior temporal lobe and right lateral Heschl’s gyrus (HG) (Liebenthal et al. 2003) while increased activations have been identified in the right anterior superior temporal sulcus and a set of regions of the bilateral middle and inferior temporal gyri (Giraud et al. 2004), the posterior part of the left superior temporal gyrus (STG) extending along the superior temporal sulcus (Dehaene-Lambertz et al. 2005) and the planum temporale and planum polare, and the superior/middle temporal gyri extending into inferior parietal and frontal cortices (Tuennerhoff and Noppeney 2016). Neurophysiological measures with EEG (electroencephalography) and MEG (magnetoencephalography) have also been employed to investigate the temporal profiles of neural responses. An electrophysiological mismatch response has been reported to occur earlier and more asymmetrically for a phonemic change than for an equivalent acoustic change (Dehaene-Lambertz et al. 2005). Prior knowledge-induced EEG enhancement and concurrent MEG reduction have been localized to the inferior frontal gyrus (IFG) and left STG (Sohoglu et al. 2012; Sohoglu and Davis 2016), with a temporal ordering such that activity in IFG was modulated before activity in lower-level sensory regions of the left STG. Using a pop-out paradigm, a positive correlation between delta band entrainment to phoneme-level features and perceived speech intelligibility has been reported (Liberto et al. 2018). Furthermore, a significant effect of prior knowledge on cortical entrainment to the temporal envelope of speech has been reported between distinct time windows in the left IFG and HG (Di Liberto et al. 2018).

A recent invasive electrocorticography study has quantified changes in the spectrotemporal tuning of ensemble neuronal activity with recordings obtained directly from human auditory cortex (Holdgraf et al. 2016). This tuning or feature representation, described as the neurons’ spectrotemporal receptive fields (STRFs), has conventionally been examined in animal models using single-unit recordings at different levels of the auditory pathway (Miller et al. 2002; Woolley et al. 2005). Based on ensemble STRFs (eSTRFs), Holdgraf and colleagues demonstrated a rapid automatic change of speech feature encoding in human auditory cortex, induced by prior experience of intact speech before subsequent presentations of degraded speech. This tuning shift has been suggested to facilitate extraction of speech-related features in stimuli and provide the physiological basis for the experience-enhanced speech pop-out phenomenon. Importantly, Holdgraf et al. (2016) quantified these effects using changes in high-frequency broadband activity, response similarity, and eSTRF tuning, and did not report a corresponding increase in low-frequency envelope-related phase locking after prior exposure. Their findings therefore support rapid feature-level plasticity, but do not necessarily imply that such plasticity must be detectable as an increase in the specific syllable-rate coherence measure used in the present study.

In the context of human speech perception, neurophysiological studies have shown that the auditory cortex tracks the dynamics of speech envelope, approximately at the syllabic rate (Ahissar et al. 2001; Lakatos et al. 2005; Luo and Poeppel 2007; Ding and Simon 2012; Kayser et al. 2015; Rimmele et al. 2015). Psychoacoustic studies have shown that the slowly varying temporal envelope of speech signal contains major acoustic cues that are important for speech intelligibility (Drullman et al. 1994; Shannon et al. 1995; Smith et al. 2002). This neural tracking activity, often referred to as “cortical entrainment” has been argued to be necessary for speech comprehension. However, its functional role remains controversial (Peelle and Davis 2012; Ding and Simon 2014; Zoefel and VanRullen 2015). Some authors believe that cortical synchronization with the low-frequency speech envelope actively constrains the transfer of information from sensory to higher-order brain regions and this synchronization with the speech envelope is essential for speech comprehension (Peelle et al. 2013; Zion Golumbic et al. 2013; Ding et al. 2014). Others have argued that the role of phase-locking brain responses may be restricted to encoding acoustic cues at the syllabic rhythm in speech (Nourski et al. 2009; Howard and Poeppel 2010; Doelling et al. 2014), and that the cortical responses are mainly driven by the physical properties of the acoustic input. Due to the concomitant changes in intelligibility and acoustic properties in speech stimuli, there has been a continuing debate about whether the brain envelope-following response mainly reflects processing of linguistic or acoustic information in speech.

By manipulating prior knowledge of spoken sentences, the effects of perceived intelligibility on cortical entrainment can be easily isolated from any acoustical changes in speech stimuli. However, counter to expectations and the behaviorally robust perceptual enhancement in speech intelligibility, none of the studies employing the pop-out paradigm have reported any significant effects of prior knowledge on brain activities phase-locked to the temporal envelope of speech (Millman et al. 2014; Holdgraf et al. 2016; Liberto et al. 2018). Notably, this includes Holdgraf et al. (2016), who reported rapid context-related changes in spectrotemporal tuning and response similarity, but also noted that an analysis quantifying the relationship between low-frequency theta phase and the speech envelope did not differ between the BEFORE and AFTER conditions. One study has reported significant cortical entrainment enhancement in the delta band (1 to 4 Hz) induced by perceptual pop-out, however rather than sustained throughout the duration of speech utterance it only emerged within overlapped time windows up to 400 ms (Di Liberto et al. 2018).

An important methodological advance has been provided by MEG work demonstrating that activity from auditory cortex can track “abstract” linguistic units, ie linguistic units that are embedded in connected speech but have no physical presence in the acoustic properties of the signal (Ding et al. 2016). When short sentences constructed with the same syntactic structure were presented in an isochronous manner, concurrent cortical tracking activity to syllable/word, phrase- and sentence-level linguistic units from participants was found. Importantly, this neural tracking activity of larger linguistic structure at phrase and sentence levels is unambiguously dissociated from encoding of acoustic cues to these units, because there are no physical phrase or sentence boundaries in the isochronous speech signal. The authors argued that an internal, grammar-based construction process must have been implemented (Ding et al. 2016).

Our recent MEG study has demonstrated the effect of spectral degradation modulated speech intelligibility on these tracking responses and investigated the underlying neural sources of the concurrent tracking responses (Meng et al. 2021). Results of this study showed that cortical entrainment—the coherence between brain activities and higher-order linguistic units that are less directly supported by acoustic cues than the syllable rate (phrases and sentences)—was reduced parametrically as a function of reduced intelligibility. In contrast, brain responses coherent to words/syllables that were accompanied by acoustic onsets were relatively insensitive to intelligibility changes. Beam-forming source localization analysis further demonstrated that the intelligibility modulated brain tracking activities were lateralized to the left hemisphere while the intelligibility insensitive word/syllable level tracking responses were bilateral. Importantly, these results indicated that brain responses are relatively insensitive to changes in intelligibility when linguistic and acoustic temporal regularities are mixed up together. This confound between acoustic and linguistic cues is inevitable in naturalistic speech and it may account for the mixed results reported in neuroimaging studies that employed naturalistic sentences for experimental stimuli.

Unlike previous studies, the experimental paradigm of Ding et al. (2016) provides a capable tool that dissociates higher-order linguistic structure from the dominant syllable-rate acoustic regularity in the speech stream and enables the assessment of intelligibility effects on neural responses at distinct timescales (syllable, phrase, and sentence). Based on this paradigm and the direct modulation from intelligibility changes established in Meng et al. (2021), we hypothesized that effects of prior knowledge would be most clearly observed in neural responses indexing higher-order linguistic structure (phrase and sentence), whereas responses at the syllable/word rate would be less sensitive to this manipulation because they are more directly supported by strong acoustic regularities in the stimulus stream. Noise-vocoding was used to render speech unintelligible while maintaining its temporal envelope (Shannon et al. 1995). Enhancement in speech intelligibility was achieved via a rapid perceptual learning process (Davis et al. 2005), which changes the perceptual experience of spectrally-degraded speech sentences from unintelligible to intelligible by pre-exposing listeners to matched clear speech. In this way, all the physical properties of the speech stimuli were identical before and after the change of intelligibility was introduced.

In addition to the reported “cerebral lateralization” of brain tracking responses to lower and more abstract levels of linguistic content in our previous MEG study (Meng et al. 2021), we also wished to characterize and contrast the underlying neural sources of the tracking responses facilitated by the perceptual pop-out effect. We specifically predicted that the hypothesized enhancement in tracking responses at the sentence- and phrase-level, driven by prior knowledge on the acoustic and linguistic information, would be more left-lateralized while the largely unchanged word-level responses remain bilateral.

## Materials and methods

### Participants

Twenty-three native speakers of English aged between 18 and 39 years old (mean 26 years old; 15 females) participated in this experiment. All participants were right-handed, with normal hearing and without any history of neurological, psychiatric, or developmental disorders (self-reported). Written informed consent was obtained from all participants under the process approved by the Human Subjects Ethics Committee of Macquarie University (approval number: 5201800226).

### Stimuli

The speech materials were synthesized using the MacinTalk text to speech synthesizer (male voice Alex, 360 words per minute, Mac OS ×10.13.4). In total, 180 four-syllable (a monosyllabic word for each syllable) English sentences were generated to form a sentence list (Supplementary Material S1). All sentences in the list followed the same syntactic structures: adjective/pronoun + noun + verb + noun. Each syllable was synthesized independently, and all the synthesized syllables (200 to 376 ms in duration) were adjusted to 320 ms by truncation or padding silence at the end. The offset of each syllable was smoothed by a 25-ms cosine window.

From the 180 sentences in the total pool, 60 (first set) were randomly selected to be presented in the unprocessed form (“natural speech”). A second set of 60 sentences were randomly selected from the remaining 120 sentences for “8 channel noise vocoding”, and the remaining set (third set) of 60 sentences were used for both “natural speech” and “8 channel noise vocoding”.

**Figure 1:**
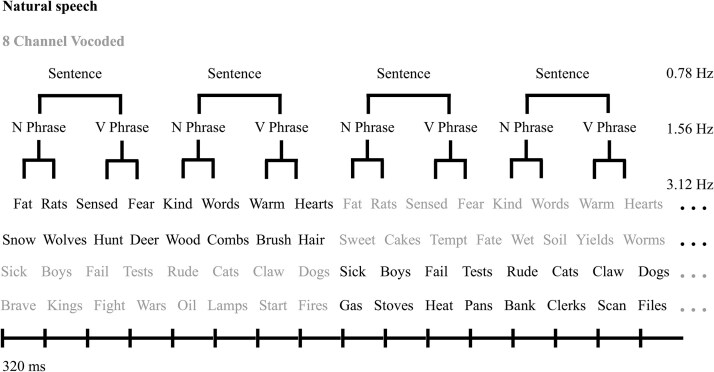
Sequences of English monosyllabic words were presented isochronously, forming phrases and sentences. Acoustic condition alternates between natural and 8 channel noise vocoding after every two sentences with either the same two sentences (matched) or a different pair (unmatched). N and V represent noun and verb, respectively. The far right column indicates the corresponding presentation rate of the linguistic structures.

With half “natural speech” and half “8 channel noise vocoding”, 12 sentences were presented in each trial. To avoid any potential artifact from the switching of acoustic conditions at the individual sentence level, every 2 sentences of the same type were grouped together so that the acoustic condition alternates at the group level (2 sentences) within a trial during presentation. The linguistic content between neighboring groups could be either matched (same sentences) or unmatched (different sentences) and the relative position of the sentence groups of different acoustic conditions also varied (Fig. 1). This produced four different experimental conditions in total. For the two unmatched conditions (“natural speech” precedes or succeeds “8 channel noise vocoding”), the first and second set of 60 sentences were both divided into two equal subsets. From each 30 sentences subset, 6 “natural speech” sentences and 6 “8 channel noise vocoding” sentences were randomly drawn for the first trials, another 6 randomly drawn from the remainder of 24, and so on to produce 5 trials of 12 sentences for each condition. For the 2 matching conditions, the same set of 60 sentences (third set) was used to generate 60 “8 channel noise vocoding” sentences and then the same operations were applied as for the mismatching conditions. This trial generation process was repeated six times to produce a total of 30 trials for each condition for the whole experiment. Over the 30 trials, each sentence was repeated 6 times. In each trial, 12 sentences (6 pairs) with alternating acoustic conditions were presented isochronously (We note that this method of stimulus construction and presentation results in speech that is significantly less intelligible than more naturalistic (non-isochronous) speech (Smith et al. 2002; Ding et al. 2014)).

Out of the 30 trials, there were 6 catch trials constructed for each condition (24 normal trials). In catch trials, 4 consecutive words selected from a random position within a trial, were replaced by 4 random words to abolish any meaningful sentence structure.

A schematic plot of the linguistic units embedded in the isochrono-usly presented syllable streams is depicted in Fig. 1 below:

### Noise vocoding

In total, 120 four-syllable sentences (including the 60 sentences for “8 channel noise vocoding” and 60 sentences for both acoustic conditions) from the sentence list were processed with noise vocoding to degrade intelligibility. Noise vocoding was implemented using custom MATLAB scripts (MATLAB and Statistics Toolbox Release 2017b, The MathWorks, Inc., Natick, Massachusetts, United States). The frequency range of 200 Hz to 22,050 Hz was divided into 8 logarithmically spaced channels using a sixth order Butterworth filter. In each frequency channel, the envelope of the speech stimulus was extracted with half-wave rectification and a low-pass filtering at 300 Hz (second order Butterworth filter). This envelope was then used to amplitude modulate white noise filtered into the same frequency channel from which the envelope was extracted. These envelope-modulated noises were then recombined over frequency channels to yield the noise-vocoded speech segments. The root-mean-square level of the noise-vocoded stimulus was normalized to match that of the original speech signal.

**Figure 2:**
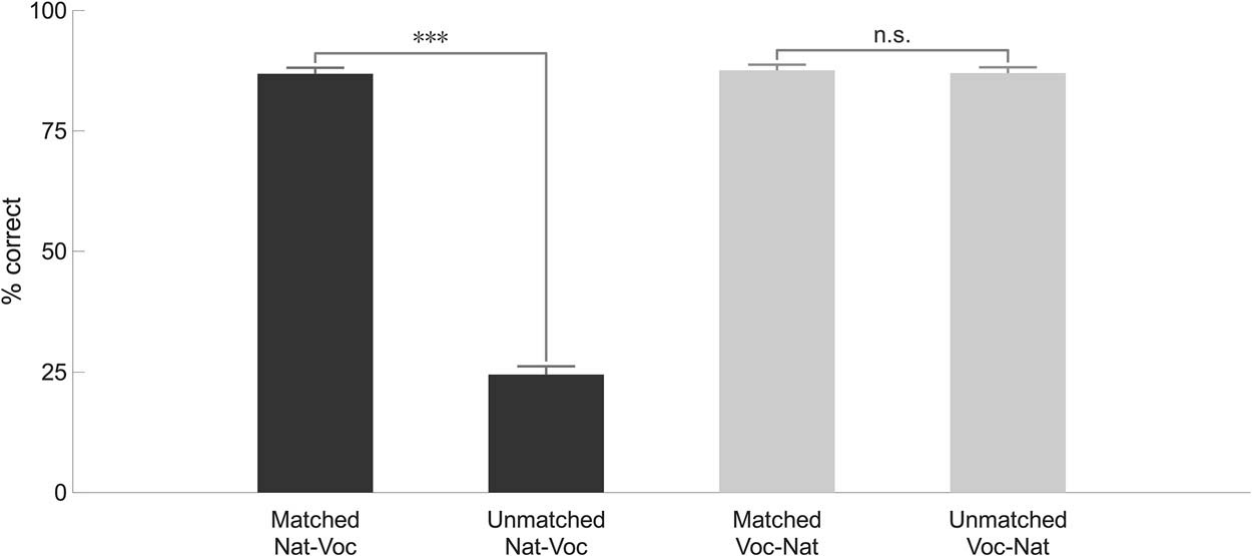
Performance of the word report task under different conditions of prior knowledge (matched speech: natural to vocoded, unmatched speech: natural to vocoded, matched speech: vocoded to natural and unmatched speech: vocoded to natural, error bars reflect standard error of mean, SEM; the stars indicate the significance levels of 0.001(^*^^*^^*^), n.s. means not significant).

To validate and quantify the effect of prior knowledge on intelligibility manipulation, a behavioral word report task was performed by a separate group of native English speakers (*n* = 30; 18 to 44 years old, mean 21 years old; 21 female). A major part of the participants data (*n* = 26) was collected online with Gorilla Experiment Builder (Anwyl-Irvine et al. 2020). In this task, participants heard two isochronously presented 4-syllable English sentences (one “natural speech” and one “8 channel noise vocoding”) at a time and were required to type only the second sentence out on the screen using a keyboard. The linguistic content (matched/unmatched) and relative position of these 2 sentences were both varied to produce 4 different experimental conditions. Several example trials from each condition were played first to each participant and then sentence pairs were presented in 4 separate blocks (30s sentence pairs/block, all conditions intermixed and evenly distributed) at a comfortable listening level. Participants indicated they had finished typing by pressing the return key, which initiated presentation of the next trial with a delay at 1.2 s. The percentage of correctly reported words is shown in Fig. 2. As expected, when natural speech precedes vocoded speech, accuracy for the matched condition was significantly higher than for the unmatched condition (*P* < 0.001, paired one-sided t test) while the accuracy for the two conditions did not differ from each other when natural speech succeeds vocoded speech (*P* = 0.625, paired one-sided t test).

### Stimulus characterization

**Figure 3:**
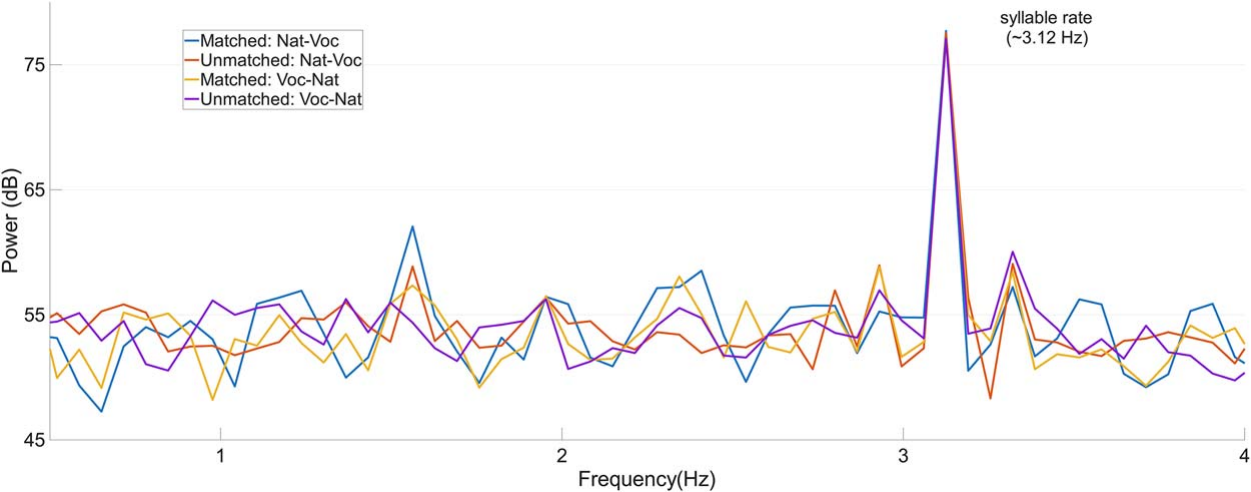
Acoustic characteristics of the speech stimuli. Power spectra of different speech stimuli. Across the four conditions, stimulus power was strongly modulated at the syllable rate (~3.12 Hz). A weaker residual component was present at the phrase rate (~1.56 Hz) in some conditions, whereas no observable residual was present at the sentence rate (~0.78 Hz) (see Fig. 1).

The slow varying temporal envelope of speech signal reflects sound intensity fluctuations and was therefore used to characterize the acoustic properties of the stimuli. The amplitude envelope of each trial (12 sentences) was extracted using half-wave rectification followed by a low-pass filtering (cut-off at 30 Hz). The mean power spectrum shown in Fig. 3 was acquired by applying a Fast Fourier Transform (FFT) to individual amplitude envelopes and then averaging within each condition. Across the four conditions, the stimulus envelopes all exhibited strong power modulation at the syllable rate. A small residual component was also present at the phrase rate in some conditions, whereas no observable acoustic residual was present at the sentence rate. Comparison of power at the phrase-frequency bin with the average power of the four neighboring frequency bins (2 bins on each side) confirmed significant phrase-rate envelope structure in the Matched: Nat-Voc, Matched: Voc-Nat, and Unmatched: Nat-Voc conditions, but not in the Unmatched: Voc-Nat condition.

### Experimental procedure

Example trials from each condition were played to each participant prior to the experiment. Experiment trials from all conditions were intermixed and evenly distributed into 4 different blocks at 75 dB sound pressure level through custom built, high fidelity insert earphones (Raicevich et al. 2010) with a flat frequency response up to 8 kHz. Participants were instructed to fix their gaze on a central cross projected to a ceiling screen and indicate whether it was a normal trial or a catch trial (containing at least one ungrammatical sentence) via a button press (with index or middle finger, right hand) at the end of each trial. The button press also initiated presentation of the next trial with a randomly-selected delay of 1.2 s, 1.4 s or 1.6 s. Each block had 24 normal trials and 6 catch trials and the trial types were presented in a pseudo-random order. A technical error resulted in 2 normal trials from each condition incorrectly marked as catch trials for 1 participant and one normal trial from a single condition (unmatched speech: Voc-Nat) was not recorded for another participant. Analysis was carried out with 22 trials across all conditions and 23 trials for that particular condition for these 2 participants.

### MEG and MRI data collection

Prior to MEG recordings, marker coil positions and head shape were measured with a pen digitizer (Polhemus Fastrack, Colchester, VT). Brain activity was recorded continuously using the KIT-Macquarie MEG160 (Model PQ1160R-N2, KIT, Kanazawa, Japan), a whole-head MEG system consisting of 160 first-order axial gradiometers with a 50-mm baseline (Kado et al. 1999; Uehara et al. 2003). MEG data was acquired with the analog filter settings as 0.03 Hz high-pass, 200 Hz low-pass, power line noise pass through and A/D convertor settings as 1,000 Hz sampling rate and 16-bit quantization precision. The measurements were carried out with participants in a supine position in a magnetically shielded room (Fujihara Co. Ltd, Tokyo, Japan). Marker coils positions were also measured before and after each recording block to quantify participants’ head movement, the displacements were all below 5 mm. The total duration of the experiment was about 45 min.

Magnetic resonance images (MRI) of the head were acquired for all 23 participants at the Macquarie University Hospital, Sydney, using a 3 Tesla Siemens Magnetom Verio scanner with a 12-channel head coil. Images were acquired using an MP-RAGE sequence (208 axial slices, TR = 2,000 ms, TE = 3.94 s, FOV = 240 mm, voxel size = 0.9 mm3, TI = 900, flip angle = 9°).

### Data analysis

MEG data analysis was performed on normal trials only (excluding the catch trials), using the open-source FieldTrip toolbox (Oostenveld et al. 2011) and custom MATLAB scripts. Offline MEG data were first filtered with a high-pass filter (0.1 Hz), a low-pass filter (30 Hz) and notch filters (50 Hz, 100 Hz, 150 Hz) and then segmented into epochs according to trial definition. To avoid excessive onset evoked responses, only the data between the start of the second sentence (or the fifth syllable if the stimulus contained no sentential structure) and the end of each trial were analyzed further (14.08 s). All data trials were down-sampled to 200 Hz prior to independent component analysis (ICA) (Makeig et al. 1996) to remove eye-blinks, eye-movements, heartbeat-related artifacts, and magnetic jumps. Components corresponding to those artifacts were identified by their spectral, topographical and time course characteristics. All cleaned trials of MEG data were kept after ICA artifact rejection.

### Sensor level analysis

The specific form of this cortical tracking of hierarchical linguistic structure has been demonstrated in a study from Zhang and Ding (Zhang and Ding 2017) as slow neural fluctuations that emerge at the beginning of the stimulus onset, rather than a series of transient responses at boundaries. Motivated by these characteristics, data analysis was carried out in the frequency domain to reveal brain activities tracking the different levels of linguistic units. Although our main hypothesis concerned the modulation of neural responses to vocoded speech by prior knowledge, the coherence analysis was performed over the analyzed portion of the whole trial rather than restricting it to vocoded segments only. This is because each trial constituted a continuous structured stream in which natural and vocoded sentence pairs alternated in a fixed rhythmic pattern, and restricting the analysis to vocoded segments alone would substantially shorten the available data and reduce the stability of low-frequency estimates, particularly at the phrase and sentence rates.

We calculated magnitude-squared coherence between the MEG recordings and an external composite signal. The composite signal consisted of the sum of 3 sinusoids at the frequencies corresponding to the presentation rates of the linguistic units of interest: syllable/word rate, phrase rate, and sentence rate. Coherence was computed separately at each frequency of interest between each MEG channel and this composite signal. Because coherence is estimated frequency by frequency, the precise phase relationships among the sinusoids in the composite signal are not critical; what matters is that the signal contains energy at the frequencies corresponding to the hypothesized linguistic regularities. We used coherence rather than spectral power or inter-trial phase coherence (ITPC) because spectral power reflects the strength of oscillatory activity within the neural signal itself but does not directly quantify its relationship to the stimulus-defined temporal structure, and ITPC similarly does not directly index coupling to an external reference signal. By contrast, coherence with the composite signal provides a direct frequency-specific measure of coupling between the neural data and the hypothesized linguistic rhythm, and also facilitates the subsequent beamforming analysis, which localizes cortical sources coherent with the same external reference signal. Magnitude-squared coherence is a frequency-domain measure of phase consistency between two signals across multiple measurements, with a normalized value between 0 and 1 at distinct frequencies. Therefore, phase relationships between these sinewaves in the composite signal can be arbitrary. MEG data trials, as well as the composite signal, were segmented into short frames of 1.28-s in length and transformed to the frequency domain using FFT with a sliding Hanning window (75% overlap, 41 frames per trial, ~ 0.78 Hz frequency resolution). These short windows were not intended to isolate complete cycles of the sentence-rate rhythm within individual segments. Rather, they were used to estimate cross-spectral density and power spectral density across many overlapping frames and trials, and the final coherence estimate therefore reflects an aggregate estimate across the full analyzed data set. The resulting frequency resolution (~0.78 Hz) matched the sentence-rate frequency of interest and its harmonically related phrase- and syllable-rate frequencies, so the goal of this procedure was not to resolve an arbitrary low-frequency spectrum but to estimate coherence at the predefined tagged frequencies embedded in the stimulus structure. Coherence was then calculated with the power spectral density of each MEG channel and the cross-spectral density between each MEG channel and the composite signal, estimated from the frequency transformed data frames.

### Source analysis

To investigate the spatial distribution of cortical areas coherent to different levels of linguistic structure, we conducted a whole-brain beamforming analysis using Dynamic Imaging of Coherent Sources (DICS) (Gross et al. 2001) which is a frequency domain, linearly constrained minimum variance beamformer (Veen et al. 1997). Source models were constructed based on each participant’s structural MRI. Cortical surface reconstruction (white-gray matter boundary) and volumetric segmentation was performed with the Freesurfer image analysis suite ((Fischl 2012); http://surfer.nmr.mgh.harvard.edu/). Cortical mesh decimation (ld factor 10 resulting in 1,002 vertices per hemisphere) and surface-based alignment was performed with SUMA—AFNI Surface Mapper (Saad and Reynolds 2012). A single shell volume conduction model (Nolte 2003) was adopted and the 2004 cortical surface vertices were used as MEG sources for the lead field calculation. For more details of the source head modeling procedure, see Li Hegner et al. (2018).

DICS was applied to the FFT transformed MEG data frames at the corresponding frequency of each linguistic unit across all intelligibility conditions. Coefficients characterizing the beamformer were computed from the cross-spectral density matrix and lead field matrix at the dominant orientation. Source level coherence images were generated by calculating coherence values between neural activity at each vertex (source point) and the composite signal using the resulting beamformer coefficients. Random coherence images were generated as the average of 100 source space coherence values calculated using the same composite signal but were randomly shuffled at each time, similar to the implementation described by Peelle et al. (2013). Cortical level group analyses were performed using cluster-based permutation test to correct for multiple comparisons (Maris and Oostenveld 2007) with a critical value of alpha = 0.01 and 2,000 random permutations. Each coherence image was contrasted with the corresponding random coherence image; the effect of immediate prior knowledge was evaluated by contrasting coherence images between the matched speech and unmatched speech conditions.

## Results

### Behavioral results

Averaged across all 30 trials under each experimental condition, accuracy rates for the vigilance task (indicating whether sentences were grammatical or not) are calculated as 68.8%, 60.0%, 72.8%, and 57.1%, respectively (summarized in Table 1). Assessed by paired two-sided t tests, accuracies were significantly higher for the matched (same speech) condition than for the unmatched (different speech) condition, whether natural speech was presented first (*P* = 0.042) or when vocoded speech was presented first (*P* < 0.001). This result reflects the greater difficulty of the “different/unmatched speech” conditions.

**Table 1:** Behavioral performance for all experimental conditions (accuracy rate mean ± SEM).

Matched: Nat-Voc	Unmatched: Nat-Voc	Matched: Voc-Nat	Unmatched: Voc-Nat
68.8 ± 2.7%	60.0 ± 3.8%	72.8 ± 2.3%	57.1 ± 2.9%

### Phase-locked responses to hierarchical linguistic structures

**Figure 4:**
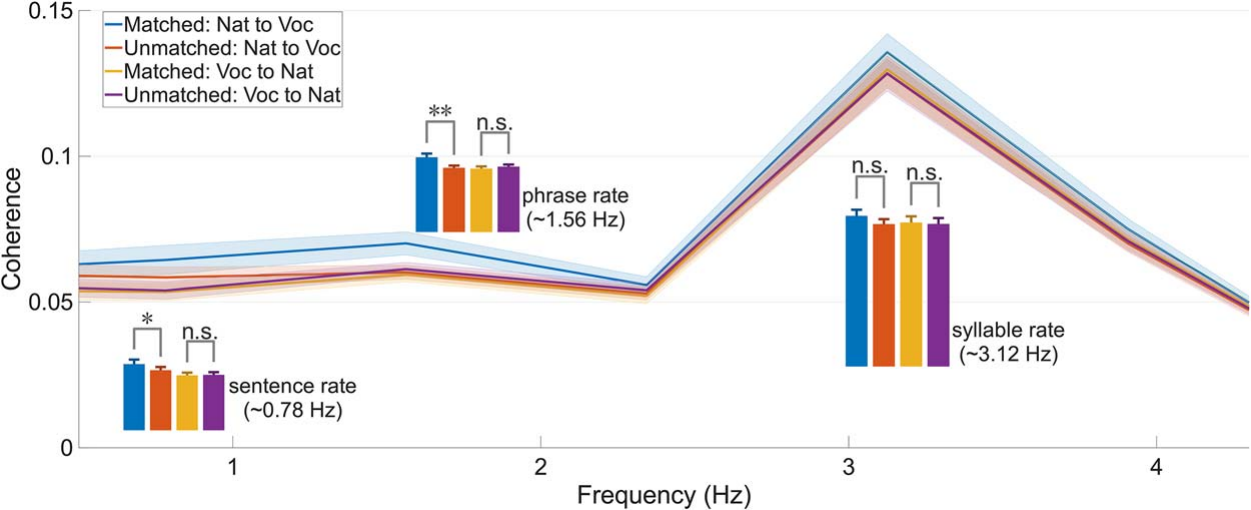
Cortical tracking responses and perceptual context. Grand-averaged MEG sensor-level coherence between the composite signal and MEG recordings (160 channels in total, averaged across channels and participants) is shown at discrete frequency bins determined by the 1.28-s analysis window (frequency resolution = 0.78125 Hz). Connecting lines are shown only to aid visualization. The shaded area indicates ± SEM. The bar plots show coherence at the tagged sentence-, phrase-, and syllable-rate bins under the four experimental conditions. Error bars indicate SEM. The stars indicate pairwise matched-versus-unmatched comparisons within each presentation order (Nat-Voc and Voc-Nat), with significance levels of *P* < 0.05(^*^) and *P* < 0.01(^*^^*^); n.s., not significant.

Magnitude-squared coherence with the composite signal calculated under each condition was grand averaged across all MEG channels as well as all participants and plotted in Fig. 4. Given the frequency resolution of the analysis (0.78125 Hz), the sensor-level coherence spectrum reflects estimates at a small number of discrete frequency bins, including those corresponding to the tagged sentence (~0.78 Hz), phrase (~1.56 Hz), and syllable (~3.12 Hz) rates. The sentence-rate response was less visually prominent than the phrase- and syllable-rate responses, and the effects reported below refer specifically to the predefined tagged frequency bins rather than to visually isolated narrowband peaks. When natural speech preceded vocoded speech, coherence in the matched condition was significantly greater than in the unmatched condition at the sentence-rate bin (*t*(22) = 2.61, *P* = 0.016, *r* = 0.49) and phrase-rate bin (*t*(22) = 3.75, *P* < 0.01, *r* = 0.63), but not at the syllable-rate bin (*t*(22) = 1.90, *P* = 0.07). Although the Nat-Voc comparison at the syllable-rate bin was numerically in the same direction as the phrase- and sentence-rate effects, this difference did not reach significance. When vocoded speech preceded natural speech, matched-versus-unmatched differences were not significant at the sentence-rate bin (*t*(22) = −0.22, *P* = 0.824), phrase-rate bin (*t*(22) = −1.05, *P* = 0.31), or syllable-rate bin (*t*(22) = 0.45, *P* = 0.66). Because the sensor-level spectrum was sampled only at discrete frequency bins, the apparent elevation of the Matched: Nat-Voc trace between the sentence- and phrase-rate bins reflects the line connecting these two sampled points and should not be interpreted as evidence for a broader low-frequency effect at unsampled frequencies.

### Cortical sources coherent to hierarchical linguistic structures

The DICS source localization results (quantified as coherence values) were overlaid on the cortical mesh of each individual participant. For visualization purposes, source space results were grand averaged and plotted on a common brain mesh generated using the Freesurfer template brain (http://surfer.nmr.mgh.harvard.edu/), segmented and processed following the procedure described in the Data Analysis section.

**Figure 5:**
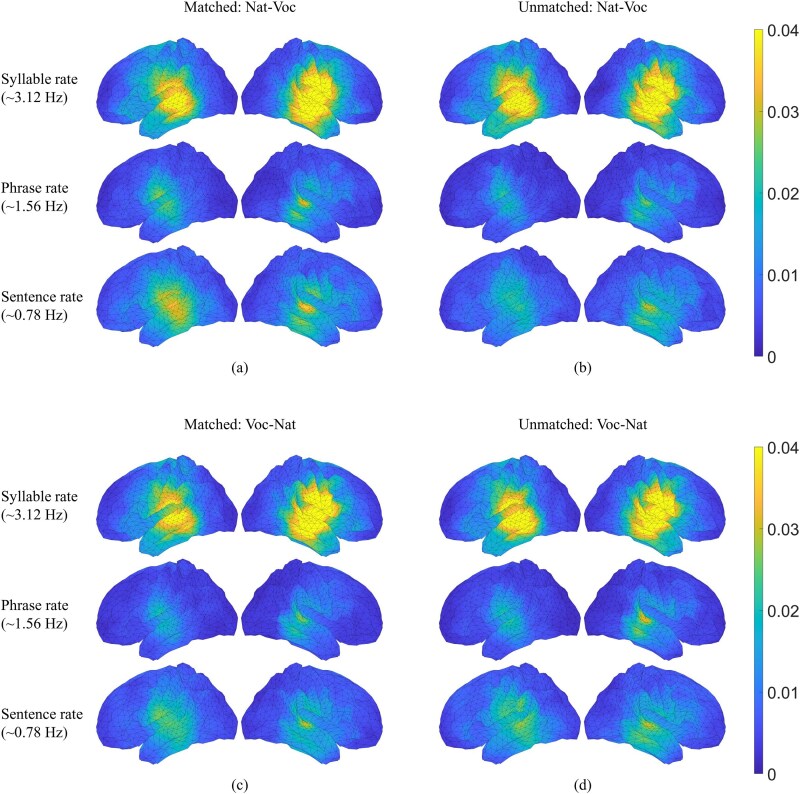
Grand mean source plots (descriptive). Grand-averaged source coherence values at the frequencies corresponding to the sentence, phrase, and syllable rates are shown for each experimental condition. These maps are descriptive visualizations of coherence distribution and are not statistical contrasts. a) Matched speech, natural speech presented prior to vocoded speech. b) Unmatched speech, natural speech presented prior to vocoded speech. c) Matched speech, vocoded speech presented prior to natural speech. d) Unmatched speech, vocoded speech presented prior to natural speech. Color bars indicate coherence values.

**Figure 6:**
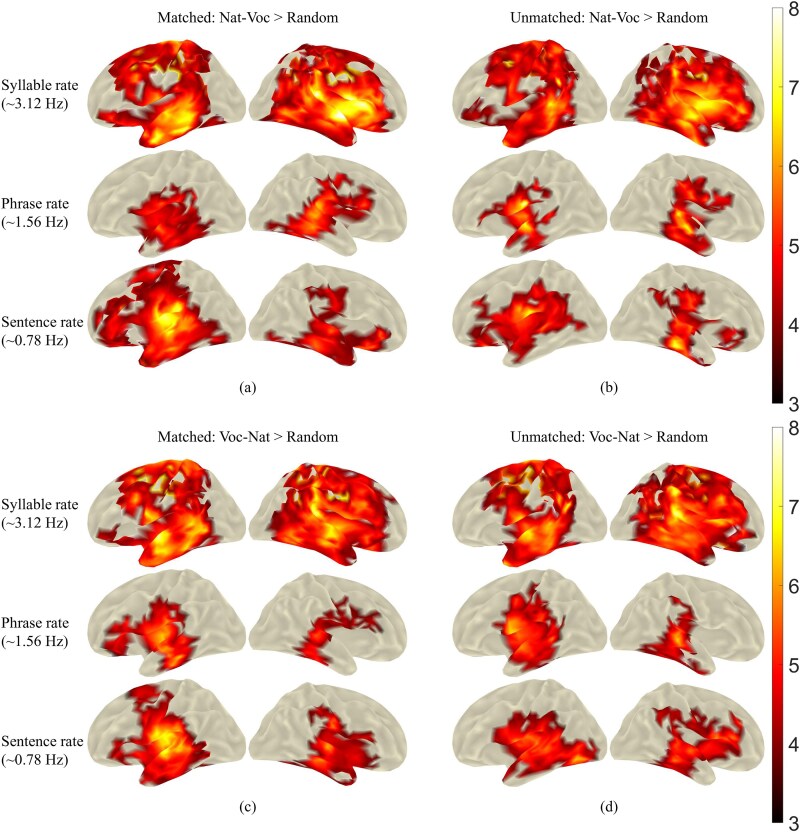
Contrasts with random coherence. Statistical contrasts between observed source coherence and random coherence are shown for each experimental condition. These maps test whether coherence at each linguistic rate is significantly greater than random coherence within each condition and do not directly test condition differences between matched and unmatched speech. a) Matched speech, natural speech presented prior to vocoded speech. b) Unmatched speech, natural speech presented prior to vocoded speech. c) Matched speech, vocoded speech presented prior to natural speech. d) Unmatched speech, vocoded speech presented prior to natural speech. Color bar indicates t values.

**Figure 7:**
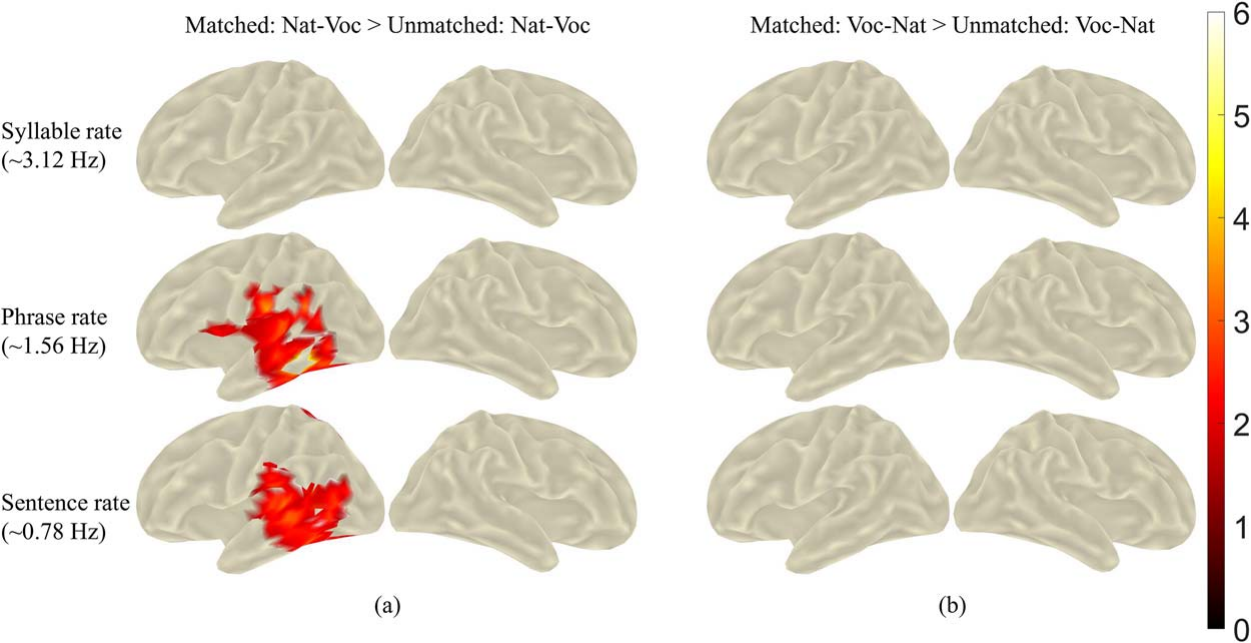
Contrasts between matched and unmatched speech. a) Cortical regions showing enhanced coherence for matched relative to the unmatched speech when natural speech was presented first. b) No significant clusters were found for the contrast between matched condition and unmatched condition when noise vocoded speech was presented prior to natural speech. Color bar indicates t values.

As a descriptive visualization, Fig. 5 shows grand mean source coherence distribution in each experimental condition and linguistic unit. Inferential statistical conclusions are based on the contrasts shown in Figs 6 and 7. Several features are worth noting prior to statistical analyses. First, mean coherence at the syllable level was bilateral and similar in size, in both hemispheres, across all experimental conditions. Second, when the natural speech was presented prior to vocoded speech, mean coherence values at the phrase and sentence levels were larger in the left hemisphere for the matched speech condition than for the unmatched speech condition. Whereas when vocoded speech was presented first, mean coherence values under matched and unmatched conditions resembled each other at the phrase and sentence levels for both hemispheres.

### Contrast with random coherence

To determine whether source-level coherence at each linguistic unit was significantly greater than a shuffled baseline, we contrasted each coherence map with a corresponding random coherence map. Unlike the descriptive plots in Fig. 5, this analysis does not directly test condition differences or hemispheric asymmetry between matched and unmatched speech. Whole-brain analyses contrasted coherence maps in each experimental condition against “random” coherence maps (calculated using shuffled composite signals—see Methods section). Results are shown in Fig. 6 using paired one-sided t tests with a sample-wise threshold of *P* < 0.0005 and a threshold of *P* < 0.0005 whole-brain cluster extent multiple comparison correction.

The results show significant coherence above the shuffled baseline at all three linguistic rates in bilateral peri-Sylvian cortices across contextual conditions.

### Contrasts between matched and unmatched speech

Whereas Fig. 6 establishes the general presence of significant tracking relative to a shuffled baseline, the contrasts in Fig. 7 directly test whether prior knowledge modulated source-level coherence by comparing matched and unmatched conditions. To examine the effect of immediate prior acoustic and linguistic context on cortical tracking activity, we conducted a whole-brain search for regions in which coherence was greater for the matched than the unmatched speech condition, separately for trials in which natural speech was presented before or after noise-vocoded speech.

As shown in Fig. 7, positive clusters were found when the same natural speech was presented before noise vocoded speech using a vertex-wise threshold of *P* < 0.05 and whole-brain cluster extent correction for multiple comparison at *P* < 0.01. The enhancements in coherence were restricted to the left hemisphere at phrase and sentence levels. Notably, no significant clusters were obtained for the syllable rate contrast (top row) or for the contrast between the matched condition and unmatched condition when the noise vocoded speech was presented prior to the natural speech.

## Discussion

A compelling neurophysiological basis for the well-studied perceptual “pop-out” effect has recently been provided from electrophysiological data recorded directly from the human auditory cortex (Holdgraf et al. 2016): Prior exposure to intact speech results in rapid and automatic enhancements in neural representations of key spectrotemporal features, which can be employed during the subsequent identification of these signals in degraded form. However, experience-driven neurophysiological effects have not been convincingly demonstrated in several studies using measures of neural entrainment (Millman et al. 2014; Holdgraf et al. 2016; Liberto et al. 2018), which are proposed to be necessary for speech comprehension. The results of the current study show that when speech intelligibility was enhanced by matched prior acoustic and linguistic knowledge, a corresponding enhancement can be obtained in MEG responses to embedded linguistic units, effects that were evident at the phrase and sentence level. Notably, because a significant phrase-rate acoustic component was also present in the unmatched Nat-Voc condition, the enhanced phrase-rate neural coherence in the matched Nat-Voc condition is unlikely to be explained solely by phrase-rate envelope structure. Statistical analyses at the source level revealed regions of greater coherence in the left temporal cortex to phrase- and sentence-level regularities. When prior information was abolished by presenting the noise-vocoded speech prior to the natural speech, no perceptual pop-out was achieved and there were also no differences in the corresponding MEG responses.

Our finding of no significant effect at the syllable level is consistent with the results of previous investigations of the perceptual “pop-out” effect that found no reliable enhancement of envelope-related low-frequency phase locking after prior knowledge (Millman et al. 2014; Holdgraf et al. 2016; Liberto et al. 2018). As discussed in our previous MEG study (Meng et al. 2021) and the EEG study from Liberto et al. (2018), one likely reason is that this response is driven largely by physical regularities present in the speech stream and is therefore less sensitive to top-down influences induced by prior knowledge.

Importantly, the absence of a significant prior-knowledge effect at the syllable rate should not be taken to mean that lower-level speech processing is uninfluenced by intelligibility. In the present paradigm, the syllable-rate coherence measure reflects phase locking to highly regular acoustic onsets imposed by the isochronous stimulus design. Because these onsets are physically marked and strongly periodic, coherence at the syllable-rate bin is likely dominated by stimulus-driven acoustic regularity and may therefore be relatively insensitive to additional top-down modulation of spectrotemporal feature processing induced by prior knowledge. In this sense, prior exposure may enhance the internal representation of speech-relevant spectrotemporal detail without necessarily producing a measurable increase in the specific syllable-rate coherence metric used here. This interpretation is also consistent with Holdgraf et al. (2016), in which robust prior-knowledge-related feature-level changes co-occurred with no detectable difference in a low-frequency envelope-related analysis.

The ubiquitous oscillatory neural activities in the brain have been proposed to provide a potential brain mechanism for deciphering the speech signal (Hickok and Poeppel 2007; Giraud and Poeppel 2012). Many neuroimaging studies have examined the low frequency entrainment to slow varying speech envelope in auditory cortex, as the putative brain process segregating linguistic units at syllable scale. However the results to date have been mixed and controversial (Peelle and Davis 2012; Ding and Simon 2014; Zoefel and VanRullen 2015). The neural response to hierarchical linguistic structures (Ding et al. 2016) provides a plausible mechanism for information integration over time (Schroeder et al. 2008; Buzsáki 2010) and enables structure building operations (Bastiaansen et al. 2009) via coupling with higher frequency neural oscillations (Sirota et al. 2003; Lakatos et al. 2005; Canolty et al. 2006). Therefore, examining this hierarchy of neural processing may provide insights into the delineating process of those controversial results from speech envelope tracking measurement, eg the perceptual “pop-out” effect facilitated by prior knowledge and top-down integration.

Our statistical maps indicate that the experience-dependent enhancement of the tracking of phrase- and sentence-level responses is associated with activity in the left cerebral hemisphere. The lateralized responses are entirely consistent with the findings reported in our recent MEG study demonstrating that the speech intelligibility modulates changes in cortical tracking responses to larger linguistic units (phrases and sentences) (Meng et al. 2021).

The issue of hemispheric specializations for speech analysis is complex (Poeppel et al. 2012) and strongly debated. Previous neuroimaging studies of the perceptual “pop-out” phenomenon have reported left hemisphere activation (Dehaene-Lambertz et al. 2005; Di Liberto et al. 2018), bilateral activation (Liebenthal et al. 2003; Giraud et al. 2004; Sohoglu et al. 2012; Sohoglu and Davis 2016; Tuennerhoff and Noppeney 2016), or no effect (Millman et al. 2014). Results of the present study also indicate that these contradictions can be partly or largely attributed to the confound between acoustic and linguistic cues in the speech stimuli employed in these studies. In contrast, when higher-order linguistic rates were much less directly supported by acoustic structure, our results showed a clear left hemisphere lateralization of cortical sources coherent to phrase- and sentence-level linguistic regularities, after the perceptual “pop-out” occurred. There has been an emerging consensus from researchers using fMRI to measure hemodynamic responses to acoustic and speech stimuli. Based on the results from a series of fMRI studies of speech comprehension, Peelle (2012) concluded that cortical lateralization depends in a roughly graded fashion on the relative amounts of linguistic processing required by the task. With a single experiment showing concurrent responses to different levels of linguistic units, our current MEG results provide a clear confirmation of this conclusion.

Since the prior-knowledge-driven enhancement in speech intelligibility relies on linguistic attributes, the cortical origins of this rapid tuning shift has been predicted to be within the auditory association areas or the non-auditory regions such as the IFG/premotor cortices (Holdgraf et al. 2016). In support of this prediction, our source analyses point to frontal-temporal origins of the top-down processes for the brain tracking responses at phrase and sentence levels, mainly encompassing ventral motor and premotor regions; and not to more anterior prefrontal executive regions. Contrary evidence has been reported in an earlier MEG study, suggesting that an early activation of left IFG initiates subsequent early speech envelope entrainment activities in left HG, superior temporal sulcus (STS), and middle temporal gyrus (MTG) (Di Liberto et al. 2018). It has been demonstrated by Meng et al. (2021) that brain responses to mixed acoustic and linguistic cues are largely driven by the acoustic cues. For studies using naturalistic speech stimuli to investigate the brain mechanism of speech envelope encoding/speech envelope entrainment, such as the one by Di Liberto and colleagues, such a confound between acoustic and linguistic cues is inevitable. For the current study, we measured and reported brain responses at higher-order linguistic rates that were much less directly supported by acoustic structure than the syllable rate, with the sentence-rate effect providing the clearest evidence for modulation without an observable acoustic envelope correlate.

As our experiment results demonstrated, the MEG responses exhibit a fast plasticity driven by prior knowledge with intelligible speech. This neural tracking activity nicely characterizes the dominant factors involved in linguistic processing, from both the bottom-up and top-down process perspectives. It therefore serves well as an objective neural marker of high-level speech processing which will be useful in basic neurolinguistics research, and also has potential clinical significance for assessment of language function after interventions for hearing loss including cochlear implantation. A growing consensus in the field of cochlear implantation is that much of the observed variability in performance may be attributable to neuroplastic changes in speech processing as a consequence of the profound sensory deprivation imposed by deafness (Wilson and Dorman 2008). The present results, taken together with the successful measurement of brain tracking responses to hierarchical linguistic units using EEG by Ding et al. (2017), provide a potentially powerful neuromarker that can be used to assess and interrogate the “compromised auditory brains” of cochlear implant recipients after the restoration of auditory inputs. Objective markers of language processing may also be useful in studies of young children and in difficult-to-test clinical populations including autism spectrum disorders.

## Supplementary Material

prior_knowledge_Meng_final_Supplementary_Material_bhag070
