# Supplementary material for: Prior exposure to speech rapidly modulates cortical processing of high-level linguistic structure
Source: Cereb Cortex. 2026 Jul 1;36(6):bhag070. doi: 10.1093/cercor/bhag070 (PMC13320234; doi:10.1093/cercor/bhag070)
Supplement: prior_knowledge_Meng_final_Supplementary_Material_bhag070 [file prior_knowledge_meng_final_supplementary_material_bhag070.docx]

Prior exposure to speech rapidly modulates cortical processing of high-level linguistic structure

Qingqing Meng^1,2,6*^, Yiwen Li Hegner^1,3,5*^, Iain Giblin^3^, Catherine McMahon^1,3,4^ and
Blake W Johnson^1,2^

^1^The HEARing CRC, Audiology, Hearing and Speech Sciences, University of Melbourne, Melbourne, Victoria, Australia

^2^Macquarie University, Department of Cognitive Science, Sydney, New South Wales, Australia

^3^Macquarie University, Department of Linguistics, Sydney, New South Wales, Australia

^4^H:EAR Centre, Macquarie University, New South Wales, Australia

^5^MEG-Center, University of Tübingen, Tübingen, Germany

^6^National Acoustic Laboratories, Australian Hearing Hub, Sydney, New South Wales, Australia

*Address correspondence to Qingqing Meng, Department of Signal Processing, National Acoustic Laboratories, Sydney, NSW 2109, Australia. Email: david.meng@nal.gov.au; Yiwen Li Hegner, MEG-Center, University of Tübingen, Tübingen, Germany. Email: yiwen.li@med.uni-tuebingen.de.

Running title: Prior knowledge enhances cortical speech processing

Supplementary Material: Sentence Stimuli

Fat rats sensed fear Kind words warm hearts Young kids close gates

Stacked shelves hold cans Long fights cause hate Flax threads hang plates

Big men drive trucks Dead sharks spout blood Their store sold jeeps

Bright flares shine light Shrewd dogs dig holes Wise cubs sip milk

Dry fur rubs skin Lean girls like jeans Four farms found cows

Sly fox stole eggs Sick boys fail tests Sharp knives cut cheese

Top chefs buy beef Rear gates stop draughts Soap suds cleanse toes

Our boss made deals Firm palms make bread Loud sounds scare moms

Two groups plant shrubs Bad smells fill town Weird clowns wear hats

All moms love kids His aunt tied shoes Her sons paint walls

New plans give hope Quiet lambs graze grass Giant bears walk trails

Large ants built nests Soft forks spill rice Drunk dudes sang tunes

Teen apes chase bugs Tree frogs stalk flies Small chicks catch grubs

Rude cats claw dogs Black skies show stars Brown bags take space

Rich cooks brew tea Tall guys flee camp Hot grills cook steaks

Fun games waste hours Grey sheep seek hills Big rocks clog roads

Pink toys please girls Iced beer costs bucks Storm floods ruin farms

Great waves wreck ships Brave kings fight wars Warm ground melts snow

Vain ears hear talk Sore eyes shed tears Keen blades slash tires

Close friends swap gifts Harsh trails sprain joints Posh wives pay bills

Horse hooves crush rocks Mad dogs bite tails Good shops pour drinks

Red lights stall cars Fine gifts please hosts Some pets climb trees

House maids scrub floors Fierce flames sear steak Snow limbs lift weights

Oil lamps start fires Snow wolves hunt deer Chrome tanks leak gas

Wood combs brush hair Sheer noise hurts ears Smart girls read books

Cold storms harm plants John’s wife bakes cakes South lane leads home

Bowled balls strike pins Blunt sticks smash glass Parched fields need help

Three teams lost games Smooth eggs hatch chicks Steep hills slow bikes

Gas stoves heat pans Cute birds build nests Deep trust bonds friends

Cheap baits halt slugs Sour food draws breath Low planes dust crops

Weak sun heats rooms Tight fists knock doors Cruel hooks catch fish

Bee stings prick arms Rough walks tax legs Thick fog blocks views

Straw hats stop sun Chopped logs choke creeks Fire doors seal smoke

Farm aids swing bats Tough spades crack slabs Bald men ride trains

Old pumps lack grease Spare keys lock halls Plump wool coats sheep

Stretched arms seize balls Iron spoon knocked floor Wall clocks tell time

Round box stores coins Bank clerks scan files Square nets grab prawns

Dried fruit tastes good Deep breaths save life Mild rain wets ground

Gold rings cause fights Small hands knead dough Slim hips twirl hoops

Shoe tread stops slips Thin ice risks lives Brick walls guard homes

Fried chips burn tongues Dark nights veil owls Dear friends send mail

Open sports draw crowds North winds bring joy Sweet cakes tempt fate

Tall trees lose leaves Lost goats scale cliffs Grown men miss youth

Cracked plates spoil food Wild pines drop cones Spiked pins pierce rags

Chilled sheets help sleep Bored dads drink beer Wet soil yields worms

Bleak seas hide crabs Bar soap cleans paws Flat screws fix lights

Back teeth hurt jaws Cool rooms keep meat Calm swells raise yachts

Huge bull jumps fence Bus tours tire guests Tight belts hold pants

Race cars dodge oil White sand covers boats Road bikes skip holes

King crabs eat shrimp Clay mugs store pens Short talks blow minds

Wine grapes have seeds Aged trains use coal Hedge plants block paths

Quick gales break kites Hard falls hurt knees Spring buds prize soil

Rose tea stains pots Used bricks fill yards Toy spoons stir cups

Hot baths treat flu Green plants feed birds Bush snakes kill mice

Axe strokes trim rope Strong light fades cloth Wide trucks move trash

Nice guys give seats Silk scarves ease throats Eight ducks cross fields

Brass clips grip notes Blue pens write words Long waits bore boys

Salt lakes ooze slime Trust funds hoard wealth Stiff brooms sweep stones

Fresh staff like work Flight crews serve lunch Steel whisks whip cream

Sun glare burns eyes Clear tape seals splits High racks hold coats
